# Optimisation and Comparison of Markerless and Marker-Based Motion Capture Methods for Hand and Finger Movement Analysis

**DOI:** 10.3390/s25041079

**Published:** 2025-02-11

**Authors:** Valentin Maggioni, Christine Azevedo-Coste, Sam Durand, François Bailly

**Affiliations:** Contrôle Artificiel de Mouvements et de Neuroprothèses Intuitives (CAMIN), Institut National de Recherche en Informatique et en Automatique (INRIA), Centre d’Université Côte d’Azur, Université de Montpellier, 34090 Montpellier, France; christine.azevedo@inria.fr (C.A.-C.); francois.bailly@inria.fr (F.B.)

**Keywords:** markerless motion capture, hand and finger kinematics, skeletal model, ecological movements

## Abstract

Ensuring the accurate tracking of hand and fingers movements is an ongoing challenge for upper limb rehabilitation assessment, as the high number of degrees of freedom and segments in the limited volume of the hand makes this a difficult task. The objective of this study is to evaluate the performance of two markerless approaches (the Leap Motion Controller and the Google MediaPipe API) in comparison to a marker-based one, and to improve the precision of the markerless methods by introducing additional data processing algorithms fusing multiple recording devices. Fifteen healthy participants were instructed to perform five distinct hand movements while being recorded by the three motion capture methods simultaneously. The captured movement data from each device was analyzed using a skeletal model of the hand through the inverse kinematics method of the OpenSim software. Finally, the root mean square errors of the angles formed by each finger segment were calculated for the markerless and marker-based motion capture methods to compare their accuracy. Our results indicate that the MediaPipe-based setup is more accurate than the Leap Motion Controller-based one (average root mean square error of 10.9° versus 14.7°), showing promising results for the use of markerless-based methods in clinical applications.

## 1. Introduction

Hand and finger motion capture is a well-established sub-field of motion capture, with applications in a variety of fields such as biomedical research and medicine (including rehabilitation aids, medical research, and biomechanical studies) [1,2], human–machine interfaces (user interfaces for disabled people, virtual reality) [3], biomechanics [4,5], and teaching and social interactions (such as sign language, emotion and behavior recognition) [6]. In particular, the assessment of hand movement performance in the context of functional assistance or rehabilitation in a clinical environment remains the topic of numerous studies, given the potential clinical benefits [7]. Marker-based optoelectronic systems, relying on the use of multiple infrared cameras and passive reflective markers placed on the body parts to be tracked, are the gold-standard for the capture of human movement, as they present remarkable accuracy with submillimetric errors [8]. However they are expensive, bulky, and require highly controlled environments and technical knowledge from the operator, hindering their use in a clinical environment. Using reflexive markers is even more complex when evaluating fine hand and finger movements [9].

Alternative markerless technologies have been extensively studied in recent years [10,11], with mixed results and applications. Early markerless motion capture techniques, which emerged before the 2000s, relied on color or contrast thresholding, background subtraction, and optical flow (which tracks the displacement of pixels over several consecutive frames) [12,13]. They paved the way for novel methods, such as the use of convolutional neural networks (CNN), which can be trained on large-scale datasets, such as the BigHand2.2M Benchmark for the hand [14]. Markerless solutions are generally more convenient and considerably less expensive than traditional motion capture systems, as they mostly rely on RGB video streams. However, their accuracy is inferior to that of marker-based solutions and can vary significantly depending on the technology. Nevertheless, these technologies are evolving at a rapid pace and their accuracy has been steadily improving over the past few years. For example, the average error per marker has dropped from 100 mm to less than 20 mm in the last ten years in the case of whole body motion capture [11].

A number of recent studies have compared markerless and marker-based methods by measuring participant movements with the two type of methods simultaneously, often focusing on the whole body, with error ranging from 40 mm to 10 mm [15,16,17,18]. A small subset of studies focusing on hand and particularly finger movements presents similarly varying degrees of error (40 to 10 mm) [19,20,21]. Among these studies, the ones with the best agreement between markerless and marker-based achieved errors of 10 mm or under for finger kinematics. For example, a study using CNN-based algorithms achieved 7.5° in joint angle error [22], but only for the movement of a single finger. Another study found errors of 11° using OpenPose (CNN-based) [23] for specific finger movements through direct comparison between the OpenPose landmarks and the markers. Alternative motion capture methods have also found some success, with one study employing infrared thermography to achieve errors under 14 mm [24]. However, the majority of these studies either focused on very specific finger movements not involving all of the hand [22], or could not record hand movements with the marker-based and markerless methods simultaneously due to the presence of physical markers interfering with the chosen markerless method [25,26]. Studies involving more dynamic finger movement [27] or hand movements involving all fingers simultaneously [20] often result in higher levels of error (20 mm for [27] and 1.5 cm for [20]). Some studies have even found the level of errors of specific markerless methods too high to be reliable in clinical trials [28,29].

To complete this comparison effort and improve markerless hand motion capture solutions, our study aims to propose a methodological framework that enhances already existing markerless methods and to quantify the error between a marker-based and two markerless methods simultaneously during ecological tasks, such as free-field movements and object grasping. The two markerless methods we selected are a CNN-based method using four webcams, “MediaPipe Hand” [1], and a combination of three Leap Motion Controllers (LMC) (Ultraleap, Bristol, UK, 2019), a commercial infrared hand motion capture device, using an additional triangulation algorithm developed by [3]. They were selected on the grounds of their affordability, their recent developments, as well as their specialisation in hand and finger tracking. They were compared to a marker-based motion capture system from Optitrack (NaturalPoint, Corvallis, OR, USA). With regard to previous studies, in which markerless and marker-based systems were evaluated on distinct measurements, for MediaPipe [26] and Leap Motion [25], this study, by using the three methods simultaneously, presents a less biased comparison between both types of approach. Additionally, the goal of this evaluation is to exploit these markerless solutions to the maximum of their capacities by using improved post-measurement data processing methods, as well as multiple recording devices. The goal is to enhance their precision and reliability in measuring variable movement types compared to the performance evaluations made in previous studies [29], and to explore whether they could be relevant in a clinical context. To this end, we developed a generic algorithm to guide the triangulation of video data from multiple cameras by selecting a specific subset of the recording cameras that maximizes the precision of the reconstructed hand movement. This approach is compatible with any video-based markerless motion capture and can be used to enhance the precision of future markerless motion studies in the field. We also compare more practical factors associated with the use of each method, as well as their potential adaptability to a clinical setting. The contributions of our work to the field of hand and finger motion capture are threefold:

- An unbiased comparison of two markerless methods to a marker-based one with simultaneous measurements for all methods for hand and finger movements.
- A novel algorithm using two criteria to select a specific subset of cameras optimizing the triangulation step of markerless motion capture, thus enhancing the precision of the estimated movement with MediaPipe. The proposed algorithm is generic, as it is compatible with all markerless video-based motion capture methods and could be reused beyond the current study.
- A quantitative study of the performances of a system using three Leap Motion Controllers to improve on the original device and complete the previous qualitative study conducted after its development [3].

## 2. Materials and Methods

### 2.1. Participants and Experimental Setup

Fifteen healthy adults (four females) volunteered for this study. All participants were thoroughly briefed about the nature of the study, potential risks and benefits, and their rights as participants. Informed consent was obtained from all participants before the experiment, in line with the Declaration of Helsinki. The only inclusion criterion was the ability to perform hand movements and object grasping, without any physical limitation. Data issued from each participant were pseudonymised to reduce any potential bias. The study was approved by INRIA’s local ethic comity (COERLE #2022-57).

**Figure 1:**
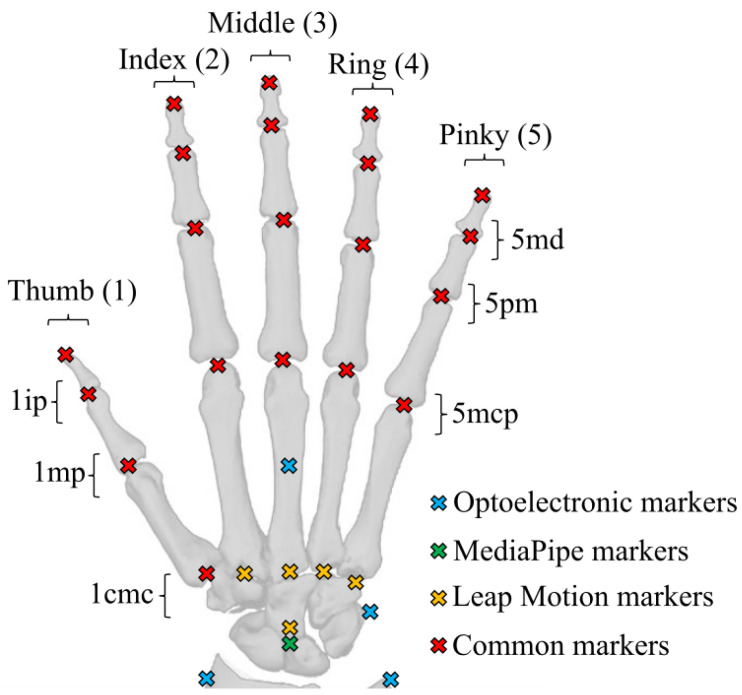
Marker sets for the optoelectronic system (blue), Leap Motion (yellow), and MediaPipe (green), and finger joint names.

The marker-based motion capture system was an Optitrack optoelectronic system, consisting of eight infrared cameras (four Flex3 and four Flex13) and 24 passive reflective markers. The hand marker set was chosen to allow the reconstruction of each segment of the hand while being as similar as possible to the ones issued by both MediaPipe and Leap Motion (Figure 1). Furthermore, we did not place three markers on each finger segment, as an overabundance of markers would impede MediaPipe’s ability to recognise the hand, an issue that has been encountered in previous studies using MediaPipe [26]. The camera placement was configured in order to maximize the tracking area and minimize occlusions. The cameras were re-calibrated as often as necessary and, by default, each half-day. The measured data were recorded at 100 Hz and reconstructed and labelled using Optitrack Motive software.

**Figure 2:**
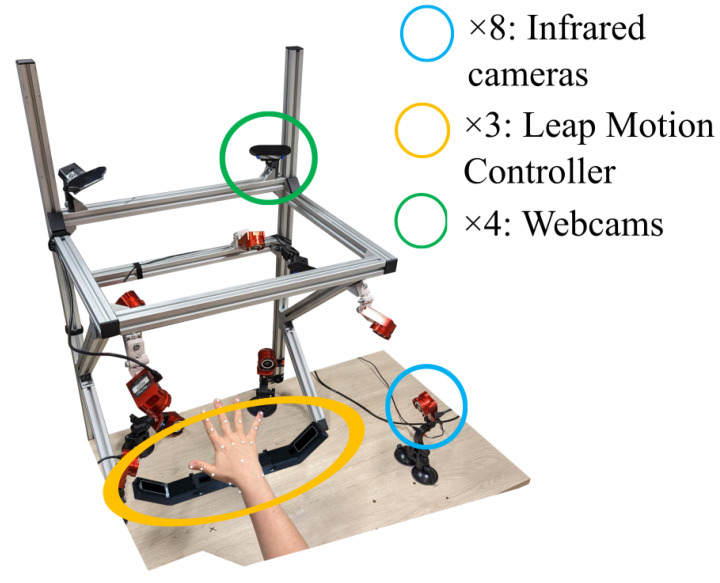
Experimental setup for the concurrent recording with the 3 motion capture methods.

The Leap Motion Controller is a 3D motion-tracking device that enables users to interact with a computer system using hand gestures in space. It relies on infrared projectors, cameras, and an advanced embedded software to track the position of the user’s hands and fingers when a hand faces the camera. Using a customized API [3], we combined three Leap Motion controllers embedded in a 3D-printed stand (Figure 2) to enhance the precision of the reconstruction and reduce occlusion issues caused by non-palm-facing hands (as the controller cannot track a hand oriented on its side or back relative to the camera). The 3D-printed stand was self-designed, with the two devices on the side being at a distance of 20 cm and a vertical angle of 45° compared to the device in the middle. The three devices were carefully aligned and calibrated to ensure optimal tracking accuracy. The collected data consist of the 3D coordinates of 28 landmarks as a function of time (Figure 1), resampled at 30 Hz. The controller was used with the 5.5.3 version of the LMC tracking software.

‘MediaPipe Hands’ is a subset of the MediaPipe open-source library developed by Google. It is a convolutional neural network (CNN)-based hand pose estimation method which allows for the tracking of hand keypoints through a regular RGB camera [1]. Similarly to the Leap Motion device, we used a set of four cameras (Logitech, C920) placed around the hand to improve reconstruction and minimise occlusions. The cameras were calibrated using a checkerboard pattern for both the intrinsic and extrinsic parameters, and were recorded at a frequency of 30 Hz. The .mp4 video files were processed offline to obtain 21 hand landmark trajectories in separated video streams (Figure 1). We used the 0.10.15 version of MediaPipe.

Each hand movement was recorded with the three measurement methods simultaneously (Figure 2). The synchronisation between them was completed by performing a typical hand gesture at the start of each recording.

### 2.2. Experimental Tasks

Each participant performed five different tasks to compare the performance of each measurement method for hand movements varying in amplitude, speed, and occlusion rates. Each hand gesture started with the palm facing down (towards the LMC) and the fingers extended. The first four hand gestures were performed with a static palm (only the fingers were moving), and the last one was performed with the whole hand moving. Each task was performed once by each participant during a trial. Each trial included 10 repetitions of the finger movements. These tasks aimed to reproduce ecological movements that one would make in their daily life, as well as to test the performance of each method when facing a wide variety of movements. The four main technical capabilities of each method that were evaluated through these tasks were (i) the precision of the detected landmarks relative to the real position of the hand, (ii) the estimated range of motion of each joint of the hand compared to the physiological maximum range of motion of the hand, (iii) the consistency of the results obtained through a given method over a joint’s entire range of motion and over multiple repetitions, and (iv) the robustness of each method in the presence of self-occlusions or external occlusions. The tasks were as follows:

- static: The hand remained static in its initial position (palm facing down, fingers extended) for the entire duration of the trial. This task presented no specific movements and served as a reference measurement.
- piano: Starting from the initial position, the participants were asked to perform consecutive finger flexion and extension, from the pinky finger to the thumb, mimicking a piano playing task. This task was used to test the precision, the measurable range of motion, and the consistency of each method over the entire range of motion of each finger individually. To this end, this task included the consecutive and independent movement of each finger, which minimised the amount of self-occlusion while making each finger as visible as possible to each system over the entire range of motion of each joint of each finger. Each finger movement was also faster in this task than in the other ones.
- flexion: Starting from the initial position, the participants were asked to repeatedly perform full finger flexion followed by a return to the initial position. This task evaluated the precision, range of motion and consistency of each method, but for synchronised movements of the finger joints, while also evaluating their robustness to a high level of self-occlusion when the fingertips are completely hidden beneath the palm.
- extension: Starting from the initial position, the participants repeatedly relaxed their fingers before extending them as much as possible. This task was used to evaluate the precision and consistency of each method, once again for syncronised movement, but this time with only a minimal amount of self-occlusion due to the fingers being close together when the hand was resting, and no self-occlusion at all when the fingers were extended. We also evaluated the maximum measurable range of motion of each finger when reaching their maximum extension.
- ball: Starting from the initial position, participants grabbed a tennis ball and repeatedly dropped it and picked it up, moving it between two marked positions on a table. This final task evaluated the performance of each method, with an emphasis on evaluating their robustness to external occlusions with the presence of the ball hiding some fingers and the underside of the palm.

### 2.3. Optoelectronic System Data

The data obtained from the eight infrared cameras were manually labelled on the Optitrack Motive software in accordance with the markerset described in (Figure 1). A first raw dataset was saved and exported to .c3d files following the labelling process, without filtering or reconstruction of the occluded data. A second dataset was then produced by filling the gaps in the landmarks trajectories, using the corresponding tools in the Motive software. The trajectory of each landmark was then filtered using the low-pass filtering tool of the software with a cut frequency setting of 6 Hz. This second dataset was exported to .c3d files.

### 2.4. Leap Motion Data

Since the three LMCs were embedded within a 3D printed support, their extrinsic calibration was completed by manually imputing their coordinates and respective orientations in a calibration file. The data were then reconstructed for each task using the API developed by [3] which uses a weighting method dependent on the orientation of the hand relative to that of the LMC. The pondered data of each device were then fused to recreate a single markerset representing the hand motion during a task. The final landmark coordinates were exported to .c3d files.

### 2.5. MediaPipe Data

The first step was to calibrate the cameras, using the Anipose Python library [30], with a video recording of a calibration checkerboard. Each camera video feed was then processed through the MediaPipe Hand algorithm, resulting in c3d files with the coordinates of the 21 hand landmarks. The MediaPipe Hand algorithm outputs 2D coordinates of each marker in a world reference frame, and a third depth coordinate relative to the position of the center of the hand [1]. However, preliminary testing revealed that this third depth coordinated was unreliable, frequently causing the estimated hand to adopt impossible poses. These initial tests also showed that the landmarks of occluded finger were not concurrent with the actual position of said fingers.

In order to improve the pose estimation accuracy, we relied on the triangulation of multiple cameras and developed an algorithm capable of identifying an optimal subset of cameras for triangulation in each trial. This subset was selected using two indicators: a reprojection error and an anatomical error. The reprojection error was defined as the distance between the coordinate of a landmark in the 2D camera frame and the coordinate of the same landmark, estimated in the 3D world frame by the triangulation operation, reprojected in the 2D camera frame. For each individual camera within each camera subset, the root mean square error (RMSE) of the reprojection error was computed both over time and over the 21 landmarks of the hand. This error was computed for each of the 10 possible subsets of cameras (by groups of two, three, and four cameras). The anatomical error was used to measure the deformations of the hand caused by a poor detection of the landmarks, leading to variations in the length of the finger segments. This error was calculated by computing the length of each finger segment over time, and the standard deviation of each segment length over the entire duration of a task. The RMSE of this standard deviation was subsequently computed over each segment and each trial to generate a global error score for each subset of cameras.

Finally, the optimal camera subset was selected for each trial as the one maximizing the number of concurrent cameras while minimizing the value of the two errors (reprojection and anatomical). The optimal camera subset selection algorithm prioritized a higher number of cameras as long as the anatomical and reprojection errors for a subset did not exceed a threshold of 10 mm for the reprojection error and 5 mm for the anatomical error, as while a higher number of cameras usually allows for a better 3D reconstruction of the movement, we still needed to avoid selecting cameras with poor measurements that would otherwise degrade the overall results.

The triangulated data from the chosen subset of cameras was finally filtered with a frequency of 6 Hz, and converted to a .c3d file.

All of the obtained .c3d data files for the optoelectronic system, Leap Motion Controller, and MediaPipe measurements were published in an open database to ensure reproducibility and the re-usability of these measurements [31].

### 2.6. OpenSim Skeletal Model

As the markersets associated with each motion capture method were different, a direct comparison between the raw landmark data was not possible. To properly compare each method and to extract results from the .c3d files, that are compatible with most biomechanical studies, we used the inverse kinematic (IK) method from OpenSim [32] to infer the finger joints values from the landmark coordinates. We used a right arm skeletal model developed by [33] that was modified to only include the hand and wrist.

Each markerset described in (Figure 1), was reproduced on the previously described model. For MediaPipe and Leap Motion, the markers were positioned at the center of each finger joint, as this is where the inverse kinematics led to the smallest error. For the optoelectronic system, the markers were positioned slightly above the joints in the direction of the back of hand, as the physical reflective markers were attached onto the dorsal skin during measurement (Figure 1). This placement is important as placing the markers at the center of the joint for the optoelectronic system would result in different hand kinematics. The aforementioned method allowed us to use three different markersets linked to the same skeletal model to accurately compare each measurement method.

To ensure that the skeletal model would fit each participant individual hand, a scaling step was necessary. The model was scaled for each participant and for each measurement method (and consequently for each markerset) using the static task measurements. The scaling was performed with the average measurement of the markers position over a period of five seconds for the static task when the hand was held still. No adjustment of the position of the model markers relatively from their original position on the bones was allowed during scaling. The scaling of each finger was only allowed alongside the finger length to account for longer or shorter finger bones. For the Leap Motion and MediaPipe models, an additional scaling was added on the proximal row to account for the positioning of the markers at the center of the palm and at the sides of the wrist.

After the scaling, we performed the IK method implemented in OpenSim to obtain the joint positions associated with each task. The IK was conducted for the entirety of each task. The weight of each marker was set to 1 for the four first proximal markers of each finger and the three first proximal markers of the thumb (Figure 1), and to 0.1 for the other markers near the palm and wrist. The weight of these markers was chosen to guarantee that the reconstructed fingers would accurately track the finger markers, while the wrist and palm markers, which are more susceptible to changes in the anatomy of each participant, held less influence on the IK.

### 2.7. Data Evaluation

In order to evaluate the error between the different methods, we computed the RMSE of the angular value of each joint between the marker-based method, acting as the reference, and each of the two markerless methods over the entirety of each trial (Figure 2). To obtain a global RMSE for a specific trial, we computed the mean of the RMSE across all joints.

As the goal of the study was to compare the ability of each method to accurately measure the position of the hand in space, only the visible markers were taken into account for the computation of the RMSE. When a marker was occluded, the RMSE of the corresponding joint was not taken into account until the maker became visible again. Moreover, if a marker was occluded for more than 50% of the task duration, the RMSE of the angle of the joint linked to the marker was not computed at all, ensuring a minimum level of visibility to include a marker in our results. The occlusion systematically concerned markers from the traditional motion capture, as the MediaPipe and Leap Motion methods inferred the position of non-visible markers. As such, the markerless methods never presented occlusions in the marker trajectories. If a physical marker from the optoelectronic system was occluded, computing an error with the markerless systems was not possible. While we could have used interpolated marker trajectories from the optoelectronic system during occlusion, we opted not to in order to avoid overestimating or underestimating the error from the markerless systems. The final rate of marker occlusion we computed corresponds to the percentage of occlusion time of a joint over the entirety of a trial. This rate of occlusion was then averaged over all joints for a participant and over all participants for each trial.

To evaluate the amplitude and amount of movement of the hand in a trial, we computed the Range of Motion (RoM) of each joint and for each trial. To obtain a global score of the amplitude of movement, the mean of the RoM was computed over all joints and for each trial.

Finally, to assess the validity of the IK method and to ensure that no additional error was introduced with it, we computed the RMSE of the distance between a marker obtained from the .c3d file of an experimental measurement and the same marker obtained from the skeletal model after the application of the IK, over the entire duration of each trial.

**Figure 3:**
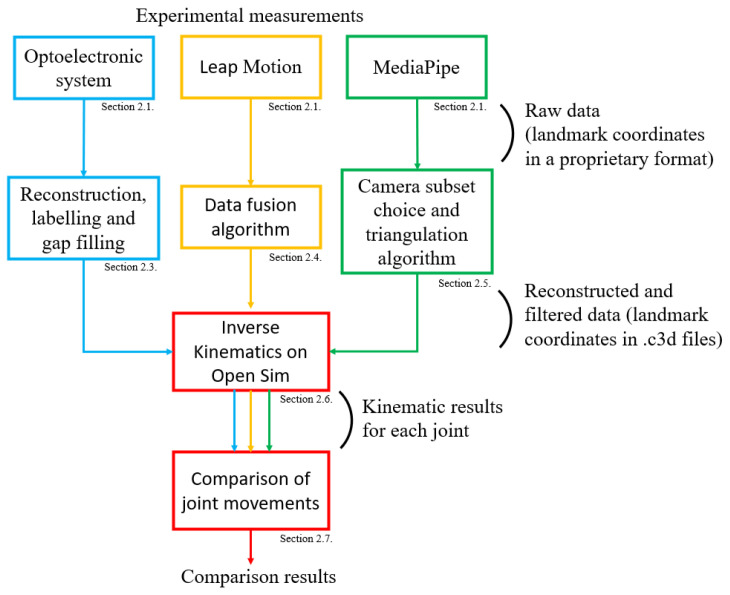
Summary of the steps of the study, from the experimental measurements to the comparison of the results, with the corresponding sections.

The global process from measurements to data processing is summarized in Figure 3.

## 3. Results

Each of the 15 participants performed five tasks (static, piano, flexion, extension, and ball) which were recorded simultaneously by the optoelectronic system, the three LMCs, and the four webcams, except for the ball task, which could not be recorded by the LMC, as the ball prevented the device from detecting the hand correctly. In total, we compiled the results from a total of 75 tasks (five tasks times 15 participants), with 210 corresponding recordings (three recordings per task for the fist four types of tasks and two recordings per ball task, one for each motion capture method used). Each recording resulted in the angle values over time of 15 joints (3 per finger) after applying the IK (Figure 1). For this entire section, the results will be presented in the format X ± Y, where X is the mean value and Y is the standard deviation.

### 3.1. RMSE Between Markerless and Marker-Based Methods

**Figure 4:**
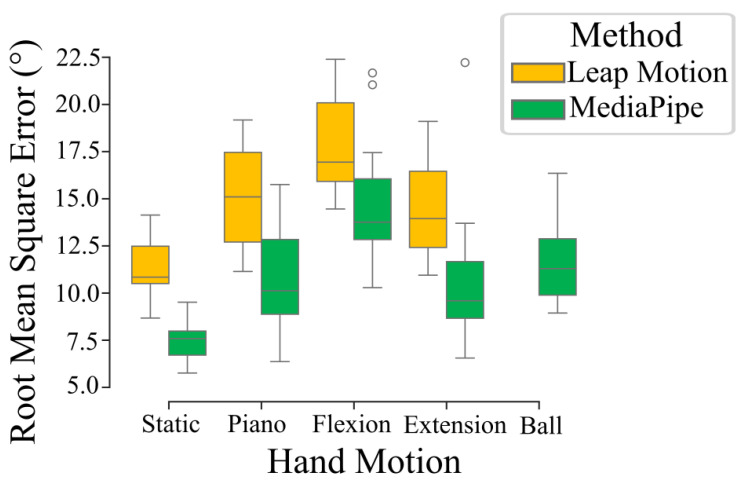
Boxplot of the root mean square error of the hand joints for the MediaPipe-based method in green and the Leap Motion method in yellow, depending on the hand motion related to each of the five experimental tasks. The values in each box are the mean of the root mean square error computed over each joint of the hand for a specific participant and a specific task. As such, each box contains 15 values (one per participant).

The means of the root mean square error between the angles of each joints obtained through the markerless methods and the marker-based were 10.9 ± 7.8° and 14.7 ± 8.6° for the MediaPipe and Leap Motion methods, respectively. The error value varied depending on the task (Figure 4), but the Leap Motion method consistently demonstrated a higher RMSE than the MediaPipe-based method. Considering the length of a finger segment between two joints, this range of error (10° to 15°) is equivalent to a distal spatial error of a few (around two to three) millimeters.

The inter-participant analysis revealed some level of variability in between measurements. The mean of the RMSE across all trials had up to 7° of differences between two distinct participants. The differentiating factors between two individual participants were the anatomy of their hands and the day on which the measurement was conducted.

Additionally, we observed joint-wise variation in the RMSE. The mean RMSE values for the majority of joints across all participants and trials were within the range 8° to 11° for the MediaPipe-based method and 10° to 14° for the Leap Motion method. Some exceptions included, for the MediaPipe-based method, the 1mp and 1ip joints (Figure 1), which had a mean RMSE of 27.3 ± 8.1° and 3.9 ± 1.6°m respectively. Similarly, the Leap Motion 1ip, 2md, and 5md joints had a mean RMSE of 2.6 ± 1.2°, 23.7 ± 7.9°, and 24.4 ± 7.5°, respectively. A summary of the mean RMSE of each joint across all trial types depending on the motion capture method is shown in Table 1.

### 3.2. Effects of Occlusions and Amplitude of Movement

**Figure 5:**
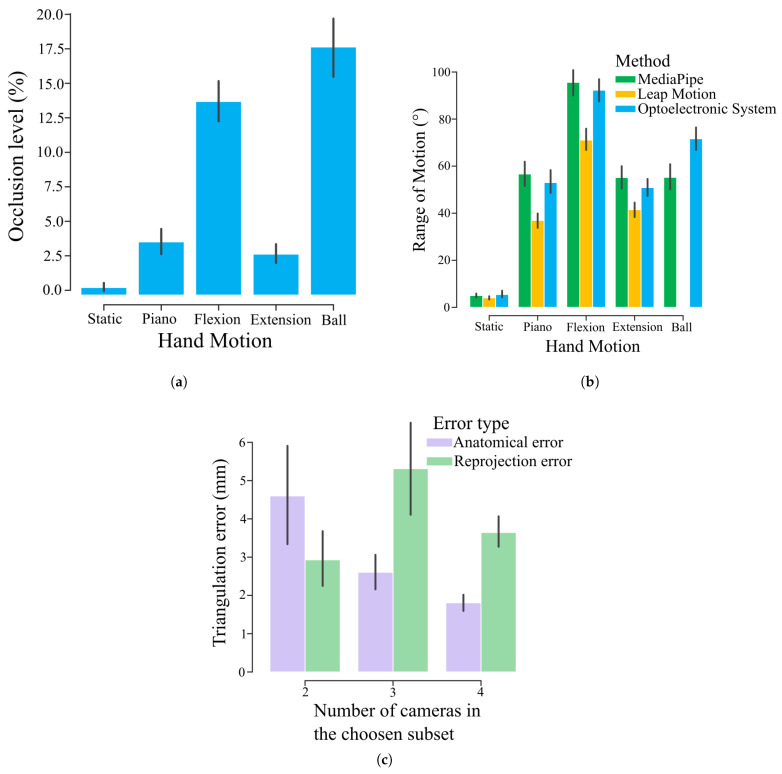
(**a**) Bar graph of the occlusion percentage of the makers depending on the hand motion. The displayed values correspond to the percentage of the time in which a marker was occluded during the entire duration of a trial, averaged over all markers of the hand and all participants. The errors bars correspond to the standard error. (**b**) Bar graph of the range of motion of the joints depending on the hand motion and measurement method with Leap Motion in yellow, MediaPipe in green, and the traditional motion capture in blue. The displayed values correspond to the difference between the maximum and minimum angle of a joint across the duration of a trial, averaged over all joints and all participants. The error bars correspond to the standard error. (**c**) Bar graph of the anatomical error (in purple) and reprojection error (in green) computed during the triangulation step of the MediaPipe-based method depending on the number of cameras in the chosen subset. The error was computed for each trial, then averaged over all trial and participants. In total, 51 trials included 4 cameras, 12 trials included 3 cameras, and 12 trials included 2 cameras. The errors bars correspond to the standard error.

The rate of marker occlusions (percentage of time during which a marker is occluded) changed extensively across trial types, reaching up to 16.6 ± 18.8% for the flexion trials and 22.3 ± 18.2% for the ball trial, despite being as low as 1.8 ± 11.2% for the static trials (Figure 5a).

As mentioned in the data processing section, only the markers with a rate of occlusion below 50% were kept in the final dataset. In total, 44 out of the 1125 marker trajectories recorded with the optoelectronic system were discarded (less than 5%). Moreover, the majority of these heavily occluded marker trajectories were situated on the wrist, as these were more easily hidden from the cameras by the palm and the rest of the hand.

To estimate the amplitude of finger movement, we computed the RoM of each joint during each trial type. Results showed a higher RoM from the flexion trials than from the other trials where the fingers were not retracted fully within the palm (Figure 5b). Moreover, we can see that the RoM from the Leap Motion measurements was lower than the RoM from the other two methods, while the optoelectronic system and MediaPipe-based method had a similar RoM, meaning that the MediaPipe-based solution was closer in movement amplitude to the optoelectronic system than the LMC.

### 3.3. Role of Multiple Cameras

For the MediaPipe-based method, the triangulation algorithm could select two to four cameras depending on the quality and consistence of the recordings. The number of selected cameras proved to have an impact on the quality of the 3D reconstruction, as measurements presented a decrease in the anatomical error with an increase in the number of chosen cameras (Figure 5c). The reprojection error, however, did not appear to be correlated to the number of cameras. The triangulation algorithm selected a full set of all four cameras 68% of the time, and a subset of three or two cameras 16% of the time each, for a total of 75 measured tasks. In 70% of the cases in which a subset of two or three cameras was selected, it was because the reprojection error of the four-camera subsets was higher than the threshold, and in the remaining 30% of cases, both the reprojection error and the anatomical error of the four-camera subset were higher than the corresponding thresholds.

### 3.4. Error of the IK Method

When computing the RMSE of the position between a marker obtained from one of the three motion capture methods, and the same marker after applying the IK, we found error values for the Leap Motion, MediaPipe, and marker-based motion capture of 2.8 ± 1.3 mm, 4.3 ± 2.3 mm, and 4.0 ± 2.9 mm, respectively, after averaging the RMSE over all markers, all participants, and all trials. This error is of the same order of magnitude as the error in position induced by the error in angle values previously computed between each method after running the IK. For the MediaPipe and marker-based motion capture methods, the obtained mean RMSE for the MCP markers (Figure 1) ranged from 5 mm to 8 mm, while ranging from 2 mm to 5 mm for the PIP, IP, and TIP markers. This higher error for the MCP markers was not observed for the Leap Motion devices, with mean RMSE ranging from 2 mm to 5 mm for all markers.

### 3.5. Comparative Analysis of the Use of Each Motion Capture Method

The specificities of each motion capture method are summarized in Table 2 and are detailed in the discussion section. In particular, it can be noted that:

The setup and calibration time of the Leap Motion controllers depend on whether the controllers are embedded in a pre-constructed structure where their positions are known, and if they need to be manually calibrated using hand recordings.

The post processing time of a recording of the optoelectronic system can take much longer (up to several hours) in specific cases with high and irregular rates of marker occlusion due to the need to manually label and reconstruct each missing marker trajectory.

While some more recent tools can assist in the partial automatization of the post-processing method of traditional motion capture, the nature of the industrial software used with the infrared cameras still implies some form of manual intervention, while the markerless methods can be entirely automated.

As seen with the previous results, the precision of the markerless methods can vary depending on the range and type of movement performed by the hand.

## 4. Discussion

### 4.1. Comparison Between Marker-Based and Markerless Motion Capture

This study proposes a direct comparison between a marker-based and two markerless methods for capturing hand and finger movements, as well as dedicated methods using multiple instances of each markerless device to improve the motion capture results. The precision of each method was assessed by measuring several hand movements with all three methods simultaneously and comparing the resulting captured hand motions.

The combination of several markerless motion capture devices is a prerequisite for the targeted precision as, in comparison, single device measurements led to errors of up to 3.5 cm (20–30°) for MediaPipe [20] and up to 38° for the LMC [21], which are much higher than the errors we obtained through the use of multiple devices. Moreover, the developed data processing algorithms used are both easily reproducible [3] and use publicly available libraries and API to facilitate the calibration and triangulation operations [30], as well as to apply the MediaPipe algorithm on already captured videos [1]. Although both markerless methods rely on the use of multiple recording devices to obtain a more precise 3D reconstruction, they remain much cheaper than an optoelectronic system (Table 2).

The two markerless methods, despite both combining multiple devices, still present a few key differences. The first one is the RoM captured for each trial type (Figure 5b), which shows that the Leap Motion-based solution has a lower RoM than the MediaPipe-based method and optoelectronic system for all the trials with dynamic finger movements (piano, flexion, and extension). The second one is that the optoelectronic system and the MediaPipe-based solution have similar RoM over all trial types. For example, for the Piano task, the optoelectronic system and the MediaPipe-based method have a mean RoM of 53° and 56°, respectively, while the LMC method has a mean RoM of 37°. On the raw c3d files, this was visible based on the fact that the LMC had more difficulty capturing the exact position of the fingers when they were below the palm during finger flexion, estimating the hand as slightly more extended than it really was.

When looking more specifically into the RMSE between the optoelectronic and markerless systems, we can observe different results depending on which error we focus on. For example, when focusing on the RMSE averaged over all joints for each trial type, we found for the LMC that the combination of the three devices resulted in a mean RMSE between the markers trajectories of the LMC compared to the optoelectronic system ranging from 10° to 20°. Meanwhile, the combination of the four webcams running MediaPipe software resulted in a mean RMSE averaged over all finger joints ranging from 8° to 13°, depending on the type of trial. This resulted in an error in position for each finger segment of a few millimeters, which is similar to the results of the other studies on hand and finger markerless capture with the lowest error values [20,28] and is lower than the errors reported for full body markerless motion capture [11,16]. If we look at the RMSE averaged over all joints and trial types, but for each participant separately, we observe that while the global RMSE for both markerless methods tend to remain between 8° and 20°, an inter-participant variability of up to 7° between trials becomes apparent. This variability could be in part due to differences in experimental conditions and participant physiology (luminosity, hand shape and size, or exact marker position, for example). However, it only corresponds to a variation of a few (1–2) mm, showing an acceptable level of repeatability. By averaging the RMSE over all participants and trial types for each individual joint, we also observed a variability in the RMSE of specific joints. In particular, the MediaPipe-based method showed more errors at the base of the thumb, corroborating similar findings in another study using MediaPipe [26], while the LMC showed higher errors at the tips of the index and of the pinky. Both methods showed little variability in all other joints.

From these results, we see that the MediaPipe-based method offers an overall more accurate reconstruction of the hand and fingers than the LMC, both for the RoM and for the RMSE with the optoelectronic system, regardless of the type of error we focus on and across all movement types and participants. Only a few specific joints proved to have a higher RMSE for the MediaPipe-based method than for the LMC method (see Table 1). The proposed method also presented lower errors of around 50% than measurements made with a single webcam (from 20–30° with a single device to 10–20° with our developed method [20]).

Additionally, the hindsight from this experimental campaign allows us to make a few recommendations for future studies involving markerless motion capture. When using video-based markerless systems, using more cameras tends to enhance the precision of the measurements, especially by reducing the anatomical error (Figure 5c). However, we observed that MediaPipe can sometimes produce poorer results for specific measurements depending on the movement or the orientation of the hand relative to the camera, which is why eliminating a camera from the final result with the specific triangulation algorithm described in this study can be beneficial. Moreover, having cameras with as many different orientations towards the hand as possible, including viewpoints from below the hand, offers more options for the camera subset selection, and thus leads to better results.

### 4.2. Validity of the Results

In order to ensure the validity of the previously discussed results, we checked at multiple stages of our study (Figure 3) that no additional errors were introduced, specifically due to the IK operation on OpenSim and to the presence of occlusions in the recordings of the optoelectronic system.

We found that the error in marker positions introduced by the IK operations (error between a marker before and after the IK) was of a similar order of magnitude (a few mm) to the RMSE between the two markerless methods and the marker-based method. Additionally, it is lower at the extremities of the fingers (2 mm to 5 mm) than at their base (5 mm to 8 mm). This difference is due to a higher difficulty in scaling the bones within the palm compared to the finger bones. In the worst case scenario, a cumulative error introduced by the IK alongside each joint of the kinematic chain of a finger could amount to up to 30 mm (for the markers after the third joint in the chain). However, the data in Table 1 show that no such scenario occurred, as the error of each joint appears independent from the rest of the kinematic chain, and only reaches values of up to around 20 to 30°. As such, the IK method does not induce errors in the positions of the markers that are significant enough to lead to an overestimation of the errors of the markerless methods.

Moreover, as we considered all marker trajectories with an occlusion rate under 50%, some markers with significant occlusion rates were still included in our results, especially for the flexion and ball trials (Figure 5a). We opted to do so to ensure a minimum level of visibility of a marker to compute the mean value of the RMSE during a sufficient number of time steps during which the marker was visible. Similarly, for the retained marker trajectory, the reason the RMSE was computed only using the time-steps where a marker was visible was to ensure that the data we used for the comparison were accurate during the post-processing steps. While the presence of occlusion is a well-known problem in marker-based motion capture, especially for finger kinematics [24,29], precise metrics on how the rate of occlusion can affect the motion capture results, such as the percentage of time during which a marker is occluded (Figure 5a) are rarely given, making a direct comparison with our results on occlusion rate impossible.

### 4.3. Limitations

Although we studied the performance of markerless motion capture on a wide range of ecological tasks, we did not include highly dynamic or more edge-case movement types (such as high-speed translations of the fingers across the measuring space [27], or tasks that require changes in the orientation of the hand relative to the recording devices). Such changes can have an impact on the measurements, as we noticed that the MediaPipe algorithm showed slightly varying results depending on the camera subset chosen by the triangulation algorithm and on the cameras’ orientation with the respect to the hand. In our future work, we will consider tackling this limitation by enhancing the camera selection algorithm for the triangulation to allow it to potentially choose multiple camera subsets within a single measurement, which will allow for dynamic adaptation to changes in the orientation of the hand and to test its validity on more dynamic and longer hand movements.

Additionally, it is important to note that markerless methods can fail to capture hand movement under specific conditions. For example, the Leap Motion devices could not detect the hand when it was holding a tennis ball between its palm and the device, making motion capture while moving around objects difficult. Moreover, it could only detect a hand when it was positioned right above the device with its palm facing down, limiting the volume of capture and the range of possible movements. For MediaPipe, the method presents the same limitations as other CNN-based methods [34]. In particular, hands with visible particularities that differ from the hands that were used to train the CNN (such as hands with too many accessories, with amputated fingers, or with orthosis) may be poorly recognized. A few other studies using MediaPipe encountered such difficulties due to the high number of physical markers they used, which prevented MediaPipe from recognizing the hand [25,26]. A possible way to avoid such limitations would be to add training data to the CNN-based methods including non-traditional hand types, such as hands with visible impairments (e.g., spasticity) that can be encountered in a clinical setting. However, the MediaPipe model cannot be retrained, as it is only downloadable as is. Accounting for such data would require the use of, e.g., transfer learning on another model.

Finally, the technologies used in this study are still in active development. As such, future updates to the MediaPipe Hand API and to the LMC could contribute to enhancing the performance of each of these two motion capture methods. Future studies evaluating the performance of these tools might be required to follow their evolution.

## 5. Conclusions

In this study, we compared the accuracy of two markerless motion capture methods for the hand and fingers with a traditional marker-based motion capture setup simultaneously. This comparison was made across five different ecological tasks with variable finger dynamics performed by 15 healthy adults. The proposed data processing methods combined with the use of multiple measurement devices allowed the markerless motion capture methods to produce results close to that of a traditional marker-based method, with finger joint errors between 8° and 20° depending on the type of movement and motion capture method used. To achieve these results, we proposed a personalized camera selection algorithm for the MediaPipe-based method and studied extensively the use of multiple LMCs for a single measurement. While both methods showed promising results, the MediaPipe API makes the CNN-based markerless method cheaper, easier to develop, more precise and easier to adapt to a clinical setting because it only requires basic webcams while offering a greater range of movements and capture volume than the Leap Motion Controllers. For our future works, we will consider testing the capabilities of our method for more specific edge-case tasks as well as investigating its use in a clinical setting, in the context of motor-impairment rehabilitation of the hand.

## Figures and Tables

**Table 1:** Table of the RMSE of each finger joint depending on the motion capture method (MP for MediaPipe and LMC for the Leap Motion Controller), averaged over all participants and all trial types.

	MP	LMC
1cmc	10.2 ± 5.6°	10.3 ± 5.4°
1mp	27.3 ± 8.1°	11.2 ± 6.1°
1ip	3.9 ± 1.6°	2.6 ± 1.2°
2mcp	11.0 ± 6.1°	13.5 ± 5.7°
2pm	14.8 ± 6.4°	12.2 ± 4.3°
2md	11.8 ± 7.0°	23.7 ± 7.9°
3mcp	9.5 ± 5.2°	18.1 ± 7.3°
3pm	8.3 ± 5.4°	8.3 ± 3.7°
3md	9.0 ± 8.3°	12.8 ± 6.4°
4mcp	10.0 ± 5.3°	19.9 ± 8.0°
4pm	9.3 ± 6.0°	10.0 ± 3.8°
4md	9.9 ± 7.1°	19.2 ± 5.2°
5mcp	10.5 ± 5.6°	19.7 ± 10.3°
5pm	8.5 ± 5.0°	13.6 ± 6.2°
5md	9.2 ± 6.3°	24.4 ± 7.5°

**Table 2:** Summary of the properties of each motion capture method studied in this paper, in the specific case of hand recording.

	Optoelectronic	MediaPipe	Leap Motion
Setup and calibration time	30 min to 1 h	10 min	1 to 20 min
Post-processing time (per recording)	10 min to 3 h	1 min	1 min
Impact of Occlusions	Increased processing time and lower accuracy	Lower accuracy	Lower accuracy
Precision (error in marker position)	under 1 mm [8]	1 to 4 mm	1 to 4 mm
Cost	From $10,000 to more than $100,000	$20 per webcam	$200 per device
Automatic processing of raw data	Partial	Complete	Complete
Acquisition volume	Entire volume between the cameras	Entire volume between the cameras	Small volume above the device with the palm necessarily facing towards the device

## Data Availability

The original data presented in the study, consisting of all of the obtained .c3d data files for the optoelectronic system, Leap Motion Controller, and MediaPipe measurements, are openly available in an open database [31].
